# Myeloid checkpoint blockade improves killing of T-acute lymphoblastic leukemia cells by an IgA2 variant of daratumumab

**DOI:** 10.3389/fimmu.2022.949140

**Published:** 2022-08-16

**Authors:** Niklas Baumann, Christian Arndt, Judith Petersen, Marta Lustig, Thies Rösner, Katja Klausz, Christian Kellner, Miriam Bultmann, Lorenz Bastian, Fotini Vogiatzi, Jeanette H. W. Leusen, Renate Burger, Denis M. Schewe, Matthias Peipp, Thomas Valerius

**Affiliations:** ^1^ Division of Stem Cell Transplantation and Immunotherapy, Department of Medicine II, Christian-Albrechts-University Kiel and University Medical Center Schleswig-Holstein, Kiel, Germany; ^2^ Division of Antibody-Based Immunotherapy, Department of Medicine II, Christian- Albrechts-University Kiel and University Medical Center Schleswig-Holstein, Kiel, Germany; ^3^ Division of Transfusion Medicine, Cell Therapeutics and Haemostaseology, University Hospital, LMU Munich, Munich, Germany; ^4^ Department of Medicine II, Christian-Albrechts-University Kiel and University Medical Center Schleswig-Holstein, Kiel, Germany; ^5^ Pediatric Hematology/Oncology, Christian-Albrechts-University Kiel and University Medical Center Schleswig-Holstein, Kiel, Germany; ^6^ Center for Translational Immunology, University Medical Center Utrecht, Utrecht, Netherlands; ^7^ Children’s Hospital, University Medical Center Magdeburg, Magdeburg, Germany

**Keywords:** T-cell acute lymphoblastic leukemia (T-ALL), CD38, daratumumab, IgA, CD47, SIRPα, immunotherapy

## Abstract

Antibody-based immunotherapy is increasingly employed to treat acute lymphoblastic leukemia (ALL) patients. Many T-ALL cells express CD38 on their surface, which can be targeted by the CD38 antibody daratumumab (DARA), approved for the treatment of multiple myeloma. Tumor cell killing by myeloid cells is relevant for the efficacy of many therapeutic antibodies and can be more efficacious with human IgA than with IgG antibodies. This is demonstrated here by investigating antibody-dependent cellular phagocytosis (ADCP) by macrophages and antibody-dependent cell-mediated cytotoxicity (ADCC) by polymorphonuclear (PMN) cells using DARA (human IgG1) and an IgA2 isotype switch variant (DARA-IgA2) against T-ALL cell lines and primary patient-derived tumor cells. ADCP and ADCC are negatively regulated by interactions between CD47 on tumor cells and signal regulatory protein alpha (SIRPα) on effector cells. In order to investigate the impact of this myeloid checkpoint on T-ALL cell killing, CD47 and glutaminyl-peptide cyclotransferase like (QPCTL) knock-out T-ALL cells were employed. QPTCL is an enzymatic posttranslational modifier of CD47 activity, which can be targeted by small molecule inhibitors. Additionally, we used an IgG2σ variant of the CD47 blocking antibody magrolimab, which is in advanced clinical development. Moreover, treatment of T-ALL cells with all-*trans* retinoic acid (ATRA) increased CD38 expression leading to further enhanced ADCP and ADCC, particularly when DARA-IgA2 was applied. These studies demonstrate that myeloid checkpoint blockade in combination with IgA2 variants of CD38 antibodies deserves further evaluation for T-ALL immunotherapy.

## Introduction

Antibody-based immunotherapy is a rapidly growing field with more than 100 antibody product approvals since 1986 and more than 50% of them during the last decade (1). The CD38 antibody daratumumab is an example of how individual antibodies can change the therapeutic landscape, in this case for multiple myeloma patients (2). For B cell precursor acute lymphoblastic leukemia (BCP-ALL), three antibody-based immunotherapeutics (rituximab, blinatumumab and inotuzumab-ozogamicin) have been approved so far (3). While antibody-based immunotherapy for patients with T cell (T)-ALL is not yet established, some monoclonal antibodies against T cell expressed antigens such as C-C chemokine receptor type 4 (mogamulizumab), CD52 (alemtuzumab), and CD30 (brentuximab-vetodine) have been approved in the treatment of other indications and are currently evaluated preclinically and clinically also in T-ALL (4, 5). The CD38 antigen is also expressed by many BCP- and T-ALL cells (6, 7), and both daratumumab and isatuximab, the second CD38 antibody approved for myeloma therapy, are being tested in clinical trials (5). Preclinical studies showed that both antibodies possess significant *in vitro* and *in vivo* activity against ALL cells with strong antibody-dependent cell-mediated cytotoxicity (ADCC) and antibody-dependent cellular phagocytosis (ADCP) effects (8–10).

Myeloid cell-mediated ADCC or ADCP can be increased by blocking myeloid checkpoint molecules – such as the CD47/signal regulatory protein alpha (SIRPα) axis (11). For example, daratumumab in combination with CD47 blockade was effective in T-ALL cell depletion *in vivo* in xenograft NSG mouse models (12), which was most likely caused by myeloid effector cells since these severely immunocompromised mice do not have T or NK cells and lack a functional complement system. The CD47 blocking antibody hu5F9-G4 (magrolimab) combined with the CD20 antibody rituximab showed promising clinical activity in advanced lymphoma patients (13). Another emerging strategy to impede CD47/SIRPα interactions is the pharmacological inhibition of glutaminyl cyclases, especially glutaminyl-peptide cyclotransferase like protein (QPCTL), which is highly expressed in tumor cells. QPCTL catalyzes the formation of pyro-glutamate, an amino acid derivative localized at the N-terminus of CD47, crucially involved in binding to SIRPα on myeloid cells (14, 15). Inhibition of glutaminyl cyclases in tumor cells by small molecules has been shown to inhibit binding of soluble SIRPα-Fc fusion proteins and to enhance myeloid cell activation against tumor cells (15–17). Antibody isotype switching from human IgG1 to IgA2 can further improve myeloid cell activation, in particular when neutrophils contribute to antibody efficacy (18). Here, we demonstrate the capacity of an IgA2 variant of daratumumab to trigger myeloid cell-mediated ADCP and ADCC against T-ALL cells when combined with CD47 blockade.

## Materials and methods

All experiments with human material were approved by the Ethics Committee of the University Medical Center Schleswig-Holstein in accordance with the Declaration of Helsinki. Healthy volunteers and patients gave written informed consent before analyses.

### Isolation of human effector cells

Polymorphonuclear granulocytes (PMN) and peripheral blood mononuclear cells (PBMC) were isolated from peripheral blood of healthy donors by density gradient centrifugation using either Polymorphprep^®^ (Progen, Heidelberg, DE) or Ficoll Paque Plus (GE Healthcare, Chicago, IL, USA), respectively, as previously described (19, 20). PBMC were then used for generation of non-polarized (M0) macrophages as described (17). Briefly, after incubation in monocyte attachment medium (PromoCell, Heidelberg, DE) for 30 min at 37°C, cells were washed three times with PBS to dispose non-adherent cells and resuspended in X-VIVO 15 medium (Lonza, Basel, CH) supplemented with 0.5% v/v penicillin/streptomycin (Gibco, Amarillo, TX, USA). After culturing for 24 h, 50 ng/ml M-CSF (PeproTech, Rocky Hill, CA, USA) were added and refreshed every 72 h at least twice before macrophages were used in ADCP experiments.

### Cell lines and patient samples

Human T-ALL cell lines HSB-2, MOLT-13, and P12-ICHIKAWA (referred to as P12) as well as CHO-S and CHO-K1 were purchased from DSMZ (German Collection of Microorganisms and Cell Cultures, Braunschweig, DE). Generation and cultivation of human CD38 transgenic CHO-K1 cells (CHO-K1-CD38^+^) was described previously (21). MOLT-13 QPCTL Knock-Out (KO) and MOLT-13 CD47 KO were generated using the CRISPR/Cas9 method. The gRNA sequences used for CD47 were ^5’^ATGCTTTGTTACTAATATGG^3’^ & ^5’^AATAGTAGCTGAGCTGATCC^3’^, and for QPCTL were ^5’^GCUUCCGAUCAAUGGGACCU^3’^ & ^5’^UAAGUGCUCCAGAGACGCUG^3’^. MOLT-13 control cells were transfected with Cas9 only (without gRNA). Primary T-ALL cells were from patients included in the ALL-BFM study 2000/2009 and described elsewhere (12).

### Antibodies and reagents

The approved CD38 antibody daratumumab (human IgG1, DARA-IgG1, clone 005, DARZALEX^®^) was from Janssen Biotech (Horsham, PA, USA). An IgA2 variant of daratumumab (DARA-IgA2) was generated *de novo* (see below). Isotype control antibodies were the EGFR antibody cetuximab (human IgG1, clone 225; Erbitux^®^), which was obtained from Merck (Kenilworth, NJ, USA), and its IgA2 variant generated as described (22). The Fc silent CD47 antibody variant 5F9-IgG2σ with V234A/G237A/P238S/H268A/V309L/A330S/P331S substitutions and the soluble SIRPα-IgG2σ protein (referred to as SIRPα-Fc) were produced as described (17, 23). Murine antibodies against human CD38 (clone HB-7), CD47 (clone CC2C6) as well as the isotype control antibody (clone MOPC-21) were from BioLegend (San Diego, CA, USA). Murine antibody against CD47 (clone B6H12) was from Thermo Fisher Scientific (Waltham, MA, USA). All-*trans* retinoic acid (ATRA) was purchased from Sigma Aldrich (St. Louis, MO, USA).

### Production and purification of DARA-IgA2

An IgA2 variant of daratumumab (DARA-IgA2) was produced based on the variable regions of daratumumab, which were *de novo* synthesized (Eurofins, Ebersberg, DE) according to the published sequences (patent WO 2017/079150 A1), and the constant region of an IgA2.0 variant of human IgA2 (22). The variable light chain (VL) sequence was cloned into the pSecTag2/Hygro C vector (coding for the kappa light chain), while the vector pCI Neo (coding for the IgA2.0 Fc heavy chain) was used for the variable heavy chain (VH). The vectors contained light chain (LC) and heavy chain (HC) secretion leaders of rituximab, respectively. For IgA2 antibody production, CHO-S cells were transiently transfected with both vectors at a VH : VL ratio of 1:1 by static electroporation using the MaxCyte STX electroporation system (MaxCyte, Gaithersburg, MD, USA) (24) according to the manufacturer’s instructions. Antibodies were purified from the supernatant by affinity chromatography using human IgA-CH1 binding camelid-derived single domain (VHH) fragments (CaptureSelect Hu IgA-CH1 Affinity Matrix; Thermo Fisher Scientific, Waltham, MA, USA). After elution with 0.1 M glycine buffer at pH 2.5 and neutralization with 1 M TRIS at pH 8, antibodies were dialyzed in PBS and subsequently applied to a size exclusion chromatography (Superdex 200 26/600 in combination with the ÄKTAprime liquid chromatography system, both from GE Lifescience, Chicago, IL, USA). High performance size exclusion chromatography (HP-SEC) was performed in PBS as mobile phase. Monomeric IgA2 containing fractions were collected and concentrated with a 100 kDa spin column (Vivaspin 20, Sartorius, Göttingen, DE). All antibodies were sterile filtered using 0.22 µm filters.

### Flow cytometric analyses

All flow cytometric analyses were performed by indirect immunofluorescence on a Navios flow cytometer (Beckman Coulter, Fullerton, CA, USA). Briefly, CD38 and CD47 specific antigen binding sites (SABC) on T-ALL cell lines and primary tumor samples were quantified using the QIFIKIT^®^ (DAKO, Glostrup, DK) according to the manufacturer’s instructions (25). The murine antibodies HB-7 for CD38 and B6H12 for CD47 were used. Antibody B6H12 detects overall cell surface levels of CD47 independent from the presence of pyro-glutamate. In contrast, expression of CD47 with N-terminal pyro-glutamate on MOLT-13 wildtype, CD47 KO and QPCTL KO cells was determined with the pyro-glutamate dependent CD47 antibody CC2C6, which recognizes the SIRPα binding site (15, 16). FITC conjugated anti-mouse IgG F(ab’)_2_ fragments were used for detection (Jackson ImmunoResearch Laboratories, West Grove, PA, USA). FITC labelled goat anti-human kappa light chain F(ab’)_2_ fragments (SouthernBiotech, Birmingham, AL, USA) were used for detection of DARA-IgG1 and –IgA2 bound on CHO-K1-CD38^+^ cells. In order to confirm the purity of the daratumumab isotype preparations, CHO-K1-CD38^+^ cells were incubated with 10 µg/ml DARA-IgG1 or DARA-IgA2 followed by FITC conjugated goat anti-human IgG or IgA F(ab’)_2_ fragments (Jackson ImmunoResearch Laboratories).

### SDS-PAGE

Purified antibodies were separated by SDS-PAGE under non-reducing and reducing conditions using a 6% and 12% acrylamide gel, respectively (Rotiphorese^®^ Gel 30, Carl Roth, Karlsruhe, DE). After a running time of 90 min at constant 120 V, gels were stained with Simply Blue Safe Stain Kit (Life Technologies, Carlsbad, CA, USA) according to the manufacturer’s instructions.

### Immunoblot

Cellular proteins were isolated by standard methods. Briefly, cells were homogenized in lysis buffer NP-40 containing PMSF and Protease Inhibitor Cocktail (all from Sigma-Aldrich, St. Louis, MO, USA). Protein amount was measured using Pierce BCA Protein Assay Kit (Thermo Fisher, Waltham, MA, USA). Proteins (60 µg per sample) were separated by SDS-PAGE under reducing conditions on a 10% acrylamide gel (Rotiphorese^®^ Gel 30, Carl Roth, Karlsruhe, DE) and subsequently transferred to PVDF membrane (BioRad Laboratories, Hercules, CA, USA). After blocking the membrane with 5% BSA in 1x TBS buffer for 1 h at room temperature, a HRP-conjugated QPCTL antibody (Santa Cruz Biotechnologies, Dallas, TX, USA, 1:500 overnight at 4°C) was used for detection. Protein loading was monitored using a rabbit monoclonal antibody against human β tubulin (abcam, Cambridge, UK, 1:2000 overnight at 4°C), followed by HRP-conjugated goat anti-rabbit IgG (Jackson ImmunoResearch, West Grove, PA, USA, 1:5000 for 1 h at room temperature). Proteins were visualized by an enhanced chemiluminescence reagent (SuperSignal West Dura, Thermo Fisher, Waltham, MA, USA).

### ADCP experiments

ADCP was measured by live-cell imaging (Incucyte^®^, Sartorius, Göttingen, DE) as previously described (23). Briefly, tumor cells were labelled with 0.5 µg/ml pHrodo for 1 h at room temperature. M0 macrophages were added at an effector-to-target cell (E:T) ratio of 1:1. ADCP in the presence of 10 µg/ml of the indicated antibodies was measured at 37°C every 40 min for 5 h. Phagocytosis was determined as red object counts per image (ROI) and analyzed using the Incucyte^®^ software (v2019B) with Top-Hat segmentation, 2 red calibrated units (RCU) as threshold and a mean intensity of ≥ 17 calibrated units (CU).

### ADCC experiments

PMN-mediated ADCC was analyzed in chromium-51 [^51^Cr] release assays as previously described (22). DARA-IgG1 or DARA-IgA2 were added at varying concentrations. Cetuximab (CTX-IgG1) and its IgA2 variant (CTX-IgA2) served as isotype controls (22). The CD47 blocking antibody hu5F9-IgG2σ was applied at 20 µg/ml. Effector cells and ^51^Cr-labelled target cells were added at a ratio of 80:1. After 3 h at 37°C, ^51^Cr release was measured in counts per minute (cpm) in a MikroBetaTrilux 1450 liquid scintillation and luminescence counter (PerkinElmer, Rodgau Jügensheim, DE). Maximal ^51^Cr release was achieved by addition of 2% v/v Triton-X 100 solution, while basal ^51^Cr release was measured in the absence of antibodies. Specific tumor cell lysis in % was calculated as follows:


$$lysis\;[\%]=\;\frac{\left(experimental\;cpm-basal\;cpm\right)}{\left(maximal\;cpm-basal\;cpm\right)}\times 100$$

### Data processing and statistical analyses

Graphical and statistical analyses were performed using GraphPad Prism 5.0 (GraphPad Prism Software, La Jolla, CA, USA). Dose-response curves were presented as means ± SEM. Statistical differences were calculated by one-way ANOVA and two-way ANOVA with Bonferroni’s *post hoc* correction and multiple comparisons. The EC50 values were reported as mean values after calculation from dose-response curves. Grouped data were presented as means ± SEM or as values of individual samples. Statistical differences were calculated by two-way ANOVA with Bonferroni’s *post hoc* correction and multiple comparisons. For primary samples, Wilcoxon matched-pairs signed rank test was used. Here, Shapiro-Wilk test showed non-normal distribution for the DARA-IgA2 group with CD47 blockade. Specifics regarding applied tests are given in the corresponding figure legend.

## Results

### The IgA2 variant of daratumumab recruits myeloid cells for T-ALL cell killing

**Figure 1:**
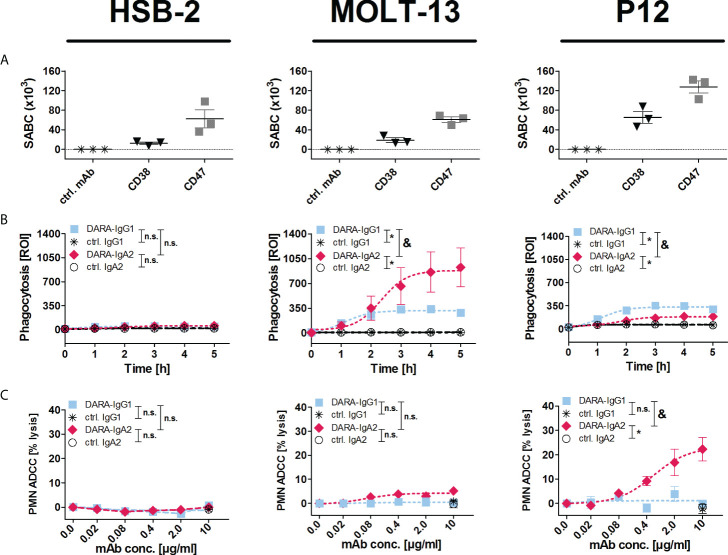
DARA-IgA2 mediates T-ALL cell killing by myeloid cells. **(A)** Expression of CD38 and CD47 on T-ALL cell lines HSB-2, MOLT-13 and P12-ICHIKAWA (P12) was quantified by indirect flow cytometry to determine the specific antigen-binding sites per cell (SABC). Mouse monoclonal antibodies against CD38 (clone HB-7) and CD47 (clone B6H12) were used at saturating concentrations (5 µg/ml) and detected with FITC-conjugated anti-mouse IgG F(ab’)_2_ fragments. The results from 3 independent experiments with means ± SEM are shown. The values of the control samples (ctrl. mAb) were subtracted from the values obtained with the antibodies. **(B)** Macrophage-mediated ADCP (E:T = 1:1) was analyzed by real-time fluorescence imaging over 5 h (300 min). Target cells were labelled with a pH-dependent red fluorescent dye (pHrodo), and phagocytosis was measured as red object counts per image (ROI) every 60 min. Antibodies were used at 10 µg/ml. Results are presented as means ± SEM of at least 3 experiments with effector cells from different donors. * indicates significant differences between DARA-IgG1 and IgG1 isotype control (ctrl. IgG1) or DARA-IgA2 and IgA2 isotype control (ctrl. IgA2) (p < 0.05 by two-way ANOVA), and & marks significant differences between DARA-IgG1 and DARA-IgA2 (p < 0.05 by two-way ANOVA). n. s., not significant. **(C)** PMN mediated ADCC (E:T = 80:1) was analyzed in 3 h ^51^Cr release assays with the indicated antibody concentrations (0-10 µg/ml) using GM-CSF (50 U/ml) stimulated PMN. Results are presented as means ± SEM of at least 3 experiments with effector cells from different donors. * indicates significant differences between DARA and isotype control (ctrl.) antibodies at the highest concentration (10 µg/ml) (p < 0.05 by one-way ANOVA), & marks significant differences between IgG1 and IgA2 (p < 0.05 by two-way ANOVA). n. s., not significant.

An IgA2 variant of daratumumab (DARA-IgA2) was produced based on the variable regions of daratumumab and the constant region of an IgA2.0 variant (22). The binding capacity of DARA-IgA2 to CD38 positive cells is similar to the original IgG1 antibody (**Figure S1**). The functionality of DARA-IgA2 against the three T-ALL cell lines HSB-2, MOLT-13 and P12 was investigated in ADCP experiments using M0 macrophages and in ADCC experiments with GM-CSF stimulated PMN. All cell lines are positive for CD38 and CD47, but expression levels vary (**Figure 1A**). No phagocytosis or PMN-mediated cytotoxicity was seen with any of the two DARA isotypes against HSB-2 (**Figure 1B, C**). In contrast, DARA-IgA2 induced significant increased ADCP of MOLT-13 cells compared to DARA-IgG1 (ROI 869.3 ± 118.0 vs. 326.1 ± 19.42, respectively; **Figure 1B**). Opposite to MOLT-13, P12 cells were only weakly phagocytosed in the presence of DARA-IgA2, while DARA-IgG1 mediated ADCP was similarly low as against MOLT-13 (**Figure 1B**). In PMN-mediated ADCC against P12 cells, DARA-IgA2 induced higher tumor cell lysis (27.11 ± 1.75% with an EC_50_ value of 0.6 µg/ml/0.004 µM) than DARA-IgG1 (1.09 ± 1.12%), no significant tumor cell lysis was observed for HSB-2 and MOLT-13 (**Figure 1C**). DARA-IgG1 did not trigger significant ADCC with PMN against any of the cell lines (**Figure 1C**).

### Genetic ablation of CD47 expression or pyro-glutamate formation increases myeloid cell mediated T-ALL killing by DARA-IgA2

**Figure 2:**
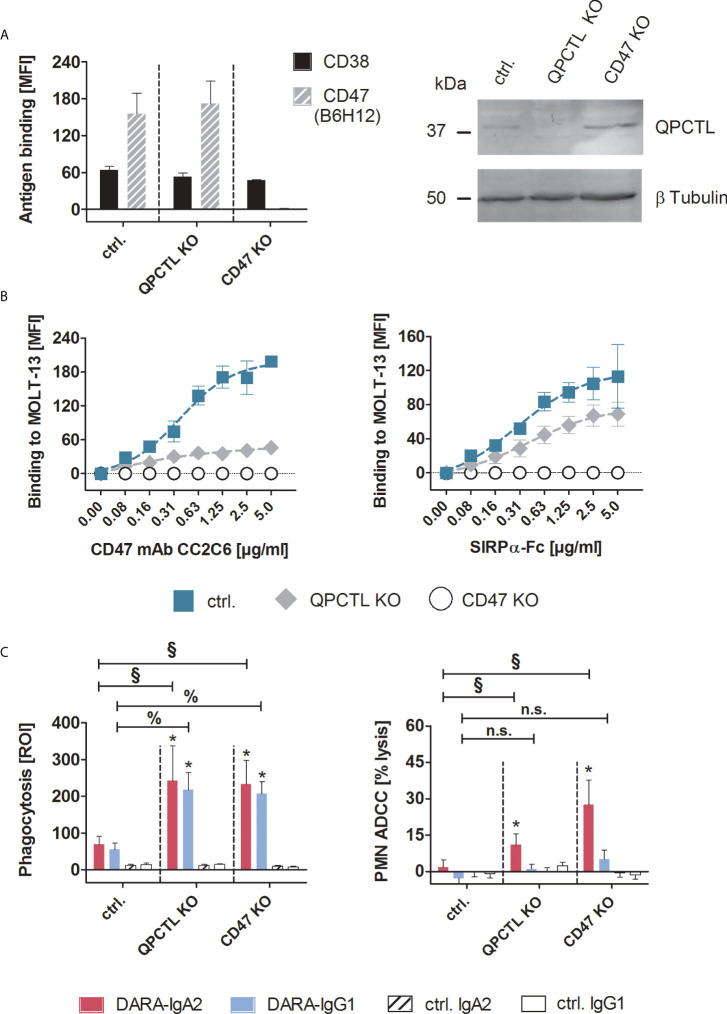
Genetic knock-out of CD47 or QPCTL improved myeloid cell-mediated T-ALL cell killing by IgG1 and IgA2 variants of daratumumab. **(A)** MOLT-13 QPCTL and CD47 knock-out (KO) cells were tested for the expression of CD38 and CD47 using antibody clones HB-7 and B6H12, respectively. The CD47 antibody B6H12 is pyro-glutamate independent and detects overall expression of the antigen. Control cells (ctrl.) were treated with Cas9 without QPCTL gRNA. Antibodies were used at saturating concentrations (5 µg/ml) and detected with FITC-conjugated goat anti-mouse Fcγ-specific F(ab)_2_ fragments (left panel, n=3). Knock-out of QPCTL was confirmed by immunoblotting (right panel), β-tubulin was used for loading control. **(B)** Binding of the pyro-glutamate dependent CD47 antibody CC2C6 (left panel) and soluble SIRPα-Fc fusion protein (right panel) on MOLT-13 control and knock-out cells was measured by indirect immunofluorescence and staining with FITC-conjugated goat anti-mouse or goat anti-human Fcγ specific F(ab)_2_ fragments. MFI values ± SEM of 3 independent experiments are shown. Background fluorescence of unlabeled cells was subtracted from each MFI value. **(C)** QPCTL KO and CD47 KO as well as control MOLT-13 cells were used as targets in macrophage-mediated ADCP (left panel) and in PMN-mediated ADCC (right panel) with DARA-IgA2 and DARA-IgG1 at 10 µg/ml. For ADCP, an E:T cell ratio of 1:1 was applied. Mean values ± SEM of the red object counts per image (ROI) after 4 h are shown (n=3 with different donors). PMN-mediated ADCC was determined by ^51^Cr release assays with GM-CSF (50 U/ml) stimulated PMN at an E:T ratio of 80:1. Percentage of mean lysis ± SEM of 4 independent experiments with effector cells from different donors are shown. * indicates significant differences between DARA and isotype control (p < 0.05 by two-way ANOVA). Significant differences between the KO variants and the control cells are indicated by § for DARA-IgA2 and by % for DARA-IgG1 (p < 0.05 by two-way ANOVA). n.s., not significant. KO, knock-out; QPCTL, glutaminyl-peptide cyclotransferase like; MFI, mean fluorescence intensity; ROI, red object count per image.

Tumor cell killing by myeloid cells can be improved by disrupting CD47/SIRPα interactions, for example with CD47 blocking antibodies (11, 26). Pharmacological inhibition of glutaminyl cyclases that catalyze the formation of pyro-glutamate on CD47 in tumor cells, has been identified as another potential strategy (12, 15–17). As a more direct approach to investigate the impact of both strategies on myeloid cell-mediated T-ALL killing, ADCP and ADCC experiments were performed with MOLT-13 cells, in which the CD47 or QPCTL genes were knocked-out by CRISPR/Cas technology. Loss of CD47 expression on cell surface in CD47 KO cells was confirmed by flow cytometry using the antibody B6H12 which detects overall cell surface levels of CD47, and knock-out of QPCTL in QPCTL KO cells was confirmed by Western blot analysis (**Figure 2A**). Importantly, the expression of CD38 remained unchanged compared to MOLT-13 control cells (**Figure 2A** left panel). QPCTL KO cells showed a 78% reduced binding of the pyro-glutamate dependent CD47 antibody CC2C6 which recognizes the SIRPα binding site (15, 16) (**Figure 2B** left), and reduced binding of soluble SIRPα-Fc (**Figure 2B** right) in comparison to control cells treated without guide RNA, confirming diminished N-terminal pyro-glutamate formation on CD47 in these cells. Neither the CD47 antibody nor soluble SIRPα-Fc showed binding to CD47 KO cells. In ADCP experiments, both QPCTL KO and CD47 KO MOLT-13 cells showed significantly enhanced phagocytosis compared to control cells, independent of the antibody isotype (**Figure 2C** left). In contrast, DARA-IgA2, but not DARA-IgG1 achieved significantly higher tumor cell lysis in ADCC experiments with CD47 KO and QPCTL KO cells compared to MOLT-13 control cells. In line with remaining SIRPα-Fc binding on QPCTL KO cells shown in **Figure 2B**, the lysis rates of QPCTL KO cells were lower compared to CD47 KO cells (**Figure 2C** right).

### DARA-IgA2 in combination with CD47 blockade enhances myeloid cell mediated T-ALL cell killing

**Figure 3:**
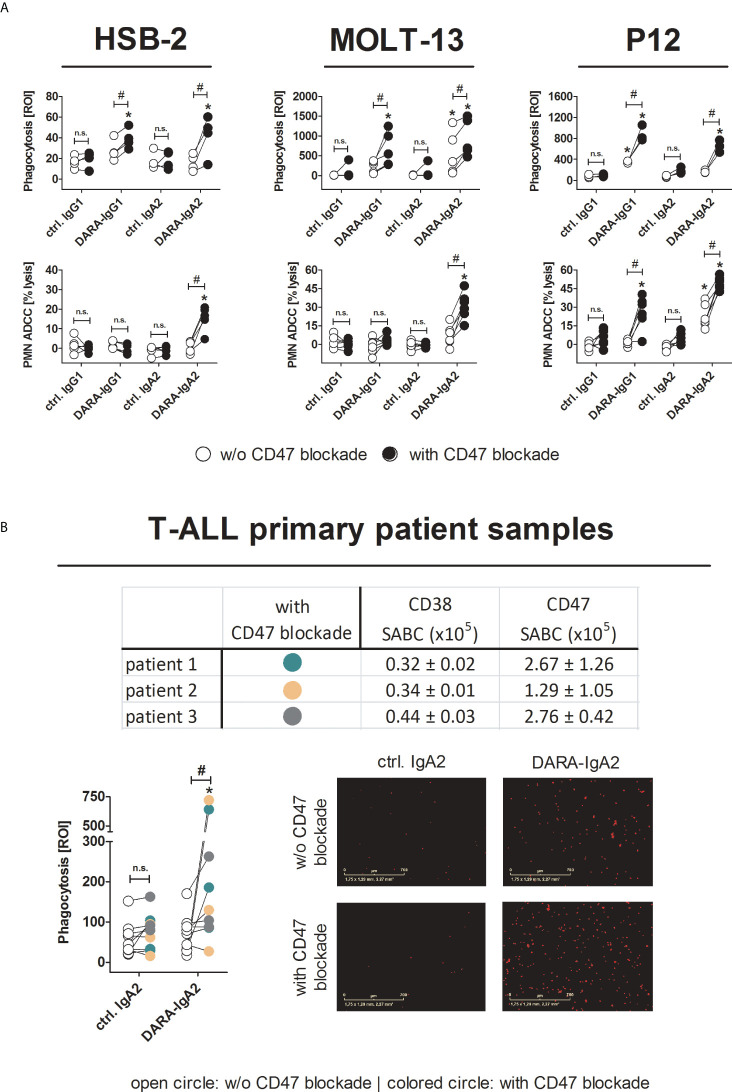
Blocking CD47/SIRPα interactions leads to efficient myeloid cell-mediated killing of T-ALL cell lines and patient samples by DARA-IgA2. **(A)** HSB-2, MOLT-13 and P12 cell lines were used as targets in ADCP (upper panel) and PMN-mediated ADCC (lower panel) assays in the absence (white circles) or presence (black circles) of the CD47 blocking antibody 5F9-IgG2σ (20 µg/ml). DARA and isotype controls were used at 10 µg/ml. For ADCP, an E:T ratio of 1:1 was applied while in ADCC assays the E:T ratio was 80:1. M0 macrophages were generated using 50 ng/ml M-CSF, PMN were activated with GM-CSF (50 U/ml). Values of at least three replicates using different donor effector cells are presented. * indicates significant differences between DARA and the respective isotype control (p < 0.05 by two-way ANOVA), # depicts significant differences between with and without CD47 blockade (p < 0.05 by two-way ANOVA). **(B)** T-ALL patient samples (n=3) were tested for expression of CD38 and CD47. Specific antigen-binding sites per cell (SABC) were quantified by CD38 antibody HB-7 and CD47 antibody B6H12 at saturating concentrations (5 µg/ml). FITC-conjugated anti-mouse IgG F(ab’)_2_ fragments were used for detection. Mean values ± SEM of three replicates are indicated in the table. ADCP assays (lower left panel) were performed in the absence or presence of the CD47 blocking antibody 5F9-IgG2σ (20 µg/ml). Patients were illustrated by different colors (color code included in table). DARA-IgA2 and isotype control were used at 10 µg/ml. An E:T cell ratio of 1:1 was applied. Shown are the red object counts per image (ROI) at maximal phagocytosis with macrophages from three different donors. Microscopic images (magnification 10x) show phagocytosed T-ALL patient cells (red dots) at the time of the highest phagocytosis rate. * indicates significant differences between DARA-IgA2 and isotype control (p < 0.05 by Wilcoxon matched-pairs signed rank test), # depicts significant differences between with and without CD47 blockade (p < 0.05 by Wilcoxon matched-pairs signed rank test. n. s., not significant.

Direct targeting of CD47 by blocking antibodies is a clinically advanced strategy to improve antibody-based immunotherapy. Daratumumab in combination with the Fc-silent CD47 blocking antibody hu5F9-IgG2σ achieved statistically significantly increases in phagocytosis of T-ALL cell lines and primary tumor cells compared to CD38 antibodies alone (**Figure 3**). Here, both DARA-IgG1 and DARA-IgA2 were effective against T-ALL cell lines (**Figure 3A** upper panel). The mean ADCP values with and without CD47 blockade for HSB-2 were 38.9 ± 4.8 ROI vs. 27.6 ± 5.1 ROI (DARA-IgG1) and 42.0 ± 9.9 ROI vs. 16.0 ± 4.0 ROI (DARA-IgA2). For MOLT-13, it was 228.7 ± 67.2 ROI vs. 669.6 ± 193.5 ROI (DARA-IgG1) and 551.0 ± 245.3 ROI vs. 939.3 ± 210.8 ROI with DARA-IgA2. Improved ADCP against P12 cells was seen with ROI values of 876.8 ± 88.3 (DARA-IgG1) and 647.7 ± 66.5 (DARA-IgA2) in the presence of the CD47 antibody, and 352.3 ± 12.7 ROI (DARA-IgG1) and 164.2 ± 14.8 ROI (DARA-IgA2) without CD47 blockade. We already demonstrated earlier that combining DARA-IgG1 with CD47 blockade leads to effective phagocytosis of primary T-ALL cells *in vitro* (12). Here, daratumumab as IgA2 in combination with CD47 blockade is also able to enhance phagocytosis of primary T-ALL cells as shown in **Figure 3B**. The tested T-ALL primary patient-derived cells all expressed CD38 and CD47 (**Figure 3B**, table). ADCP with DARA-IgA2 achieved mean ROI values of 249.4 ± 84.8 with CD47 blockade vs. 69.5 ± 15.8 without CD47 blockade and thus could be enhanced more than 3.5-fold. Phagocytosis of primary patient cells is illustrated (red dots) in the microscopic images (**Figure 3B**, right). In PMN-mediated ADCC against T-ALL cell lines, significant cytotoxicity was seen with the IgA2 isotype of daratumumab when it was combined with the CD47 blocking antibody (**Figure 3A**, lower panel). The mean lysis rates were 14.2 ± 3.4% for HSB-2 and 31.1 ± 4.5% for MOLT-13 cells. CD47 blockade resulted in significant lysis of P12 cells with both isotypes: 25.8 ± 5.5% with DARA-IgG1 and an increase from 22.7 ± 3.9% to 49.4 ± 2.1% with DARA-IgA2. Importantly, the CD47 antibody alone did not trigger significant ADCP or ADCC.

### Treatment of HSB-2 cells with ATRA enhances effector functions of daratumumab variants in combination with CD47 blockade

**Figure 4:**
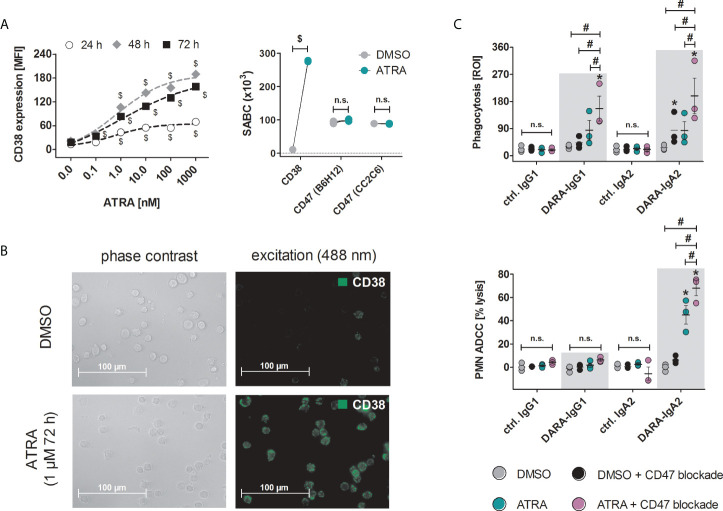
Treatment of HSB-2 cells with all-*trans* retinoic acid (ATRA) enhances CD38 expression and DARA-IgA2 mediated tumor cell killing by myeloid cells in combination with CD47 blockade. **(A)** Concentration and time dependent increase of CD38 expression on T-ALL cell line HSB-2 after treatment with ATRA at indicated concentrations for 24 h, 48 h and 72 h (left panel). CD38, CD47 expression and determination of the specific antigen-binding sites per cell (SABC, right panel) after treatment with 1 µM ATRA for 72 h were quantified using QIFIKIT. For both CD38 stainings, antibody HB-7 at saturating concentration (5 µg/ml) was used, while for CD47 staining, both B6H12 and CC2C6 antibodies were used. FITC-conjugated goat anti-mouse IgG F(ab’)_2_ fragments as secondary reagent was used. Values of three independent experiments are shown. $ depicts significant differences between ATRA and DMSO control (p < 0.05 by two-way ANOVA in both panels). **(B)** Microscopic images of HSB-2 cells stained for CD38 with antibody HB-7 and FITC-conjugated goat anti-mouse IgG F(ab’)_2_ fragments (40x magnification). **(C)** HSB-2 cells were treated with 1 µM ATRA or DMSO for 72 h and were then used in ADCP (upper panel) or PMN-mediated ADCC (lower panel) experiments. Daratumumab variants and ctrl. antibodies were applied at 10 µg/ml while CD47 blockade antibody 5F9-IgG2σ was used at 20 µg/ml. Values of three (ADCP and ADCC) different donors are depicted. * indicates significant differences between DARA and the respective isotype control (p < 0.05 by two-way ANOVA), # indicates significant differences between ATRA + CD47 blockade versus ATRA alone or CD47 blockade alone (p < 0.05 by two-way ANOVA). n. s., not significant.

Both IgG1 and IgA2 variants of daratumumab had marginal, if any, effects on ADCP or ADCC against T-ALL cells with low CD38 expression, such as the HSB-2 cell line with less than 12.4 ± 2.6 x10^3^ SABC (**Figure 1**). All-*trans* retinoic acid (ATRA) has been shown to increase the expression of CD38 on multiple myeloma and acute myeloid leukemia cells (27, 28). Treatment of HSB-2 cells with ATRA resulted in a dose- and time-dependent increase in CD38 antibody binding indicating an increase in CD38 expression, which reached its maximum with 1 µM ATRA after 48 h to 72 h (**Figure 4A**, left). The following experiments were performed with HSB-2 cells pre-treated with 1 µM ATRA for 72 h yielding a 25.1-fold increase of CD38 expression on the plasma membrane compared to DMSO treated control cells (**Figure 4A**, right; **Figure 4B**). In contrast to CD38, CD47 expression (as measured with CD47 antibody B6H12) and pyro-glutamate formation on CD47 (analyzed by CD47 antibody CC2C6) were not altered after ATRA treatment (**Figure 4A**). Additionally, binding of the CD47 blocking antibody 5F9-IgG2σ was also not influenced by ATRA treatment (data not shown). Combination of ATRA pre-treatment of HSB-2 cells and CD47 blockade resulted in significantly enhanced phagocytosis with both DARA-IgG1 and DARA-IgA2 compared to DMSO treated cells with CD47 blockade and ATRA treated cells. For DARA-IgG1, ROI values of 156.4 ± 41.4 with ATRA + CD47 blockade were achieved vs. 85.2 ± 32.2 with ATRA alone and 43.4 ± 11.7 DMSO with CD47 blockade alone (**Figure 4C**, top). Similar results were obtained with DARA-IgA2: ROI values were 198.75 ± 58.9 with ATRA pre-treatment and CD47 blockade vs. 83.5 ± 29.8 for ATRA alone and 85.2 ± 30.6 with DMSO + CD47 blockade alone. In contrast, significant PMN-mediated cytotoxicity against ATRA pretreated HSB-2 cells was only achieved with the IgA2 isotype of daratumumab (up to 45.1 ± 7.9% specific tumor cell lysis vs 6.2 ± 1.9% lysis with CD47 blockade alone) and was increased to 68.0 ± 6.4% after combining ATRA treatment and CD47 blockade. Daratumumab as IgG1 was not able to induce ADCC by PMN under these conditions (**Figure 4C**, bottom).

## Discussion

In this study, we investigated the efficiency of a novel human IgA2 variant of the CD38 antibody daratumumab to mediate T-ALL cell killing by myeloid cells. Interestingly, P12 cells were lysed efficiently by PMN, while in macrophage mediated ADCP experiments, tumor cell phagocytosis was rather weak. In contrast to P12 cells, MOLT-13 cells were efficiently phagocytosed by macrophages, but PMN mediated ADCC was not observed. Myeloid cells can express different inhibitory immune checkpoint molecules such as SIRPα, Siglecs, LILRBs (29) and additional ones are probably still undiscovered. Potentially, different expression levels of immune checkpoint ligands on tumor cells and concomitantly differential expression patterns of inhibitory receptors on myeloid cells impact tumor cell killing by different effector cell populations. Moreover, also the ratio between activating and inhibitory signals can influence the tumor cell killing (30). However, our results demonstrate that DARA-IgA2 is more efficient in activating PMN for T-ALL cell killing compared to the clinically available IgG1 antibody, especially in combination with CD47 blockade. Similar observations have been reported comparing IgG1 and IgA antibodies against other target antigens such as EpCAM, EGFR, HER2, GD2, HLA class II, CD20, CD30 and carcinoembryonic antigen (18). Neutrophils can express all three classes of IgG receptors – FcγRI (CD64), FcγRIIa (CD32a) and FcγRIIIb (CD16b) – and the IgA binding receptor FcαRI (CD89). Activation of FcαRI by clustered IgA as its natural ligand or by bispecific antibodies has been demonstrated to effectively trigger intracellular signaling (e.g. ERK activation), leading to high ADCC activity of PMN (31).

IgA2-mediated ADCP against T-ALL cell lines HSB-2, MOLT-13 and P12 and primary patient samples was significantly enhanced blocking the CD47/SIRPα axis through the genetic ablation of either CD47 or QPCTL, or by the use of a CD47 blocking antibody. However, in contrast to CD47 knock-out cells, the genetic ablation of QPCTL did not cause complete binding reduction of soluble SIRPα-Fc. The SIRPα-Fc fusion protein used here is a truncated version and the higher avidity compared to wildtype SIRPα-Fc has to be considered (32).

Notably, phagocytosis of primary T-ALL cells was quite heterogeneous, which was not due to differences in the levels of CD38 or CD47 expression, since they were similar. However, activity of the macrophages seemed to be donor- related (**Figure S2**), which e. g. may be related to alloreactivity (33) since primary T-ALL cells and macrophages were not from the same donors.

Myeloid checkpoint inhibition by blockade of CD47/SIRPα interactions is a clinically advanced approach to improve antibody-based cancer immunotherapy (11). A phase I/II study with the CD47 antibody magrolimab (hu5F9-G4) in combination with the CD20 antibody rituximab revealed mild toxicities and demonstrated encouraging clinical responses in patients with advanced non-Hodgkin’s lymphomas (13). Pre-clinical studies already showed that combining daratumumab and CD47/SIRPα blockade could be a promising therapeutic approach for T-ALL and multiple myeloma (12, 34). In our study, we used an Fc-silent IgG2σ variant of hu5F9 to investigate the impact of myeloid checkpoint blockade on CD38 antibody mediated T-ALL cell cytotoxicity. The ability to enhance myeloid cell activation by addition of the CD47 blocking antibody was observed with both, macrophages and neutrophils, and with both isotypes, DARA-IgG1 and DARA-IgA2.

Overall, the extent of antibody-dependent cytotoxicity seems to correlate with the CD38 expression, at least partially. We observed elevated CD38 expression and increased killing of HSB-2 cells upon treatment with ATRA, especially when combined with CD47 blockade. Similarly, treatment of myeloma cells with ATRA has been shown before to increase CD38 expression and improve efficacy of daratumumab *in vitro* and *in vivo* (27). The addition of ATRA to daratumumab in the treatment of patients with daratumumab-refractory multiple myeloma was safe and had some but limited activity (35). ATRA in combination with daratumumab, preferably as an IgA2 antibody, and CD47 blockade is an interesting approach for T-ALL treatment and deserves further evaluation. However, the clinical development of IgA antibodies is currently hampered by difficulties in establishing relevant *in vivo* models. Since mice do not express a functional IgA receptor (18), approaches using human CD89 transgenic mice (36, 37) and patient-derived ALL xenograft (PDX) models (38) may be valuable to make relevant progress. Myeloid cells constitute an important part of the immune cell infiltrate in ALL patients (39), suggesting that improved myeloid cell recruitment by CD38 antibodies of the IgA isotype may enhance the efficacy of antibody-based therapeutic approaches in these patients.

## Data availability statement

The original contributions presented in the study are included in the article/**Supplementary Material**. Further inquiries can be directed to the corresponding author.

## Ethics statement

The studies involving human participants were reviewed and approved by Ethik-Kommision Medizinische Fakultät der Christian-Albrechts-Universität zu Kiel. The patients/participants provided their written informed consent to participate in this study.

## Author contributions

NB, CA, JP, and ML performed the experiments and data/graph presentation. NB, CA, ML, and TR analysed the data. KK, CK, and MB, LB, FV provided essential reagents/tools. NB, RB, and TV wrote the manuscript; JL, DS, MP and TV conceived and designed the research. TV supervised the study. All authors critically revised the manuscript and approved submission.

## Funding

These studies were supported by an intramural grant from the University of Kiel to TR, by research grants from the Stiftung Deutsche Krebshilfe to CK and DMS (70113524, 70113533) and by research grants from the Deutsche Forschungsgemeinschaft (KFO 5010) to TV and DMS.

## Conflict of interest

The authors declare that the research was conducted in the absence of any commercial or financial relationships that could be construed as a potential conflict of interest.

## Publisher’s note

All claims expressed in this article are solely those of the authors and do not necessarily represent those of their affiliated organizations, or those of the publisher, the editors and the reviewers. Any product that may be evaluated in this article, or claim that may be made by its manufacturer, is not guaranteed or endorsed by the publisher.

## Supplementary material

Supplementary Figure S1 Biochemical characterization of an IgA2 version of daratumumab (DARA-IgA2). **(A)** HP-SEC profile of the IgA2 variant of DARA showing the purity of the IgA2 antibody preparation after production in CHO-S cells and purification via affinity chromatography. **(B)** DARA-IgA2 antibody preparations were separated by SDS-PAGE and gels were stained with Coomassie blue. One specific band at approx. 150 kDa was detected under non-reducing conditions (left panel) representing the complete IgA2 antibody. Under reducing conditions (right panel), two specific bands representing the heavy chain (HC) at 50 kDa and the light chain (LC) at 25 kDa. **(C)** Binding of saturating concentrations (10 µg/ml) of IgG1 and IgA2 variants of DARA to human CD38 transfected CHO-K1 cells was detected by flow cytometry using FITC-labelled anti-human IgG or IgA heavy chain-specific secondary antibodies. The MFI values ± SEM of three experiments are shown. **(D)** Dose-dependent binding to CD38 transfected CHO-K1 cells of DARA-IgG1 and DARA-IgA2 variants at increasing concentrations was analyzed by indirect immunofluorescence using an anti-human kappa light chain-directed FITC-labelled secondary antibody. Results are presented as MFI ± SEM from 3 independent experiments. MFI, mean fluorescence intensity; mAU, milli absorbance units; ctrl., control; mAb, monoclonal antibody.

Supplementary Figure S2 M0 donor dependency on ADCP of primary T-ALL cells. ADCP of primary T-ALL cells was performed in the absence or presence of the CD47 blocking antibody 5F9-IgG2σ (20 µg/ml). Patients were illustrated by different colors. DARA-IgA2 and isotype control were used at 10 µg/ml. An E:T cell ratio of 1:1 was applied. Shown are the red object counts per image (ROI) at maximal phagocytosis. * indicates significant differences between DARA-IgA2 and isotype control, # depicts significant differences between with and without CD47 blockade (p < 0.05 by two-way ANOVA). n.s., not significant.
